# Supplementary material for: Differential Expression of Immune Genes in the Rhipicephalus microplus Gut in Response to Theileria equi Infection
Source: Pathogens. 2022 Dec 6;11(12):1478. doi: 10.3390/pathogens11121478 (PMC9782190; doi:10.3390/pathogens11121478)
Supplement: Supplementary file 1 [file pathogens-11-01478-s001.zip › pathogens-2043749-supplementary.pdf]

Table S1: Primers used in the present study that target signaling pathway components with participation in the immune response of ticks.

**Gene**	**Database (access)**	**Primer-fw (5'-3 ')**	**Primer-Rv (5'-3 ')**	**Functional notation**	**Reference**
40S ribosomal S3a	GenBank [XM_037430639	GGACGACCGATGGCTACCT	TGAGTTGATTGGCGCACTTCT	Reference gene	[13] Rosa et al., 2016
β-tubulin	GenBank [CK179480]	AACATGGTGCCCTTCCCACG	GCAGCCATCATGTTCTTTGC	Reference gene	[24] Nijhof et al., 2009
Toll receptor	GenBank [KF828744]	CATCATTCGTGTGCAGTGTG	ACCAGTTGCAATGACGTCAC	NF-κB/Toll pathway	[13] Rosa et al., 2016
Toll 18Wheeler	GenBank [KF828745]	CAGAACCTGGACATCAATGC	CTTGAGGATACTGACCATCC	NF-κB/Toll pathway	[13] Rosa et al., 2016
MyD88	GenBank [KF828746]	CTGTCAAAGACGAGAACGAG	TGGAATCGCTAGACATGCTG	NF-κB/Toll pathway	[13] Rosa et al., 2016
TOLLIP	GenBank [KF828747]	CATCTGGCACCCACTTTCAG	GACCCATGGGAGCTCTTATC	NF-κB/Toll pathway	[13] Rosa et al., 2016
ECSIT	GenBank [KF828753]	CCCCAAACTCAGCGATATTC	CTTGCACTCATTGTCGGTTG	NF-κB/Toll pathway	[13] Rosa et al., 2016
TRAF	GenBank [KF828780]	AATGGCGTCAACACACTACG	TCATTTCTGTCAGCGTGTGC	NF-κB/Toll pathway	[13] Rosa et al., 2016
Cactin	GenBank [KF828752]	CCCACTGGTTAAATGGGAAG	CCCATCAATCAACAGTGTGC	NF-κB/Toll pathway	[13] Rosa et al., 2016
Tube	GenBank [KF828748]	ATGAAACACCACGGTTCTGG	TGTGCCTTTGTACACGACTC	NF-κB/Toll pathway	[13] Rosa et al., 2016
Pelle	GenBank [KF828749	GTTCCAACCATGTGAAGAGC	GTATTCACAGCAGTCTTCGG	NF-κB/Toll pathway	[13] Rosa et al., 2016
Pellino	GenBank [KF828750]	GGCGGAGTAAATTCTTCCTG	GTGTACGAAATGGAGTGCTG	NF-κB/Toll pathway	[13] Rosa et al., 2016
SARM	GenBank [KF828751]	CCATACGCGGTTTGAACCAG	GACATCGAGGCTCTTGTTCC	NF-κB/Toll pathway	[13] Rosa et al., 2016
NFKIRAS	GenBank [XM_037427340]	ACCCACCTCCAAGCAAATC	GCTTAATGTCGTGGGTTGTG	NF-κB/Toll pathway	[17] Paulino et al., 2021
Cactus (IκB)	GenBank [KF828754]	CAGTGTGCGAGAGATGTATG	CTTTAATGACCGCGATGTGC	NF-κB/Toll pathway	[13] Rosa et al., 2016
Dorsal	GenBank [KF828755]	CATATCGGGTACATCCACAC	GGCTTGTAAAGGTGCATGTC	NF-κB/Toll pathway	[14] Capelli-Peixoto et al., 2017
PGRP	GenBank [KF828741]	TCGCAGAGAATGCGCTACTG	TTCGTGTGAGCGCCTTCTGC	IMD pathway	[13] Rosa et al., 2016
Bendless	GenBank [KF828762]	GTACGGTGCTATTGTCCATC	CATTGCGTATTGCCTTGTCC	IMD pathway	[13] Rosa et al., 2016
UEV1a	GenBank [KF828763]	ATGTCTGACGAACGGGAACG	TCATGCCAGTCCAGTGAGTC	IMD pathway	[13] Rosa et al., 2016

EFFETE	GenBank [KF828764]	CCACGCGAAT CTATCATCCC	TCGACAGCAG CACTTTGGAG	IMD pathway	[13] Rosa et al., 2016
IAP2	GenBank [KF828765]	CGGAATGACA ACTGAACTGG	CCAAGAACCT GTAGAAGCTC	IMD pathway	[13] Rosa et al., 2016
TAK1	GenBank [FG301710]	CCGTGTATTG GTACAGAGTG	GTAGCAGAAA CTACACCACC	IMD pathway	[13] Rosa et al., 2016
TAB2	GenBank [KF828757]	CCAACAGCTT CGAACACAAG	GCAGGGTCTT TAGTTCTTCC	IMD pathway	[13] Rosa et al., 2016
POSH	GenBank [KF828766]	CTGATGAAGC GACTCACAGC	AGCTGTCCAC GAAACTTGGG	IMD pathway	[13] Rosa et al., 2016
IKK-α	GenBank [KF828758]	GCATTATTGC ACTACTCGCC	CAGAGCAGGA ATCTTCGAAC	IMD pathway	[13] Rosa et al., 2016
IKK-γ	GenBank [KF828759]	TCACGGTGCA GGATATACAG	GCAGAGCATC TTGATCTCTC	IMD pathway	[13] Rosa et al., 2016
Caspar	GenBank [KF828756]	GTATTCAGCA GCGAAATGGC	GTTGCTTATCC TTCTCCTGG	IMD pathway	[13] Rosa et al., 2016
Caudal	GenBank [KF828761]	CACCTTCTCAG AGTCTCAGC	GTCCGATGTTC ATTGCCAGC	IMD pathway	[13] Rosa et al., 2016
Relish	GenBank [KF828760]	GCACGACAGC CAATTATTCG	CTCTTGCTCAG CAGAAGAAG	IMD pathway	[13] Rosa et al., 2016
Hemipterous (MKK)	GenBank [KF828767]	CGATGCATCC GTGATCGTAG	CCAGGTGTTTC TTGGAACCG	JNK pathway	[13] Rosa et al., 2016
Basket (JNK)	GenBank [KF828768]	CAAGGCAAGC AACATTGAGC	TTCATGGGAG GAACTGTGAC	JNK pathway	[13] Rosa et al., 2016
JRA	GenBank [KF828769]	TGACCCTGGA CTTGAACAGC	AGCTGGAGCA TGTTCAGGTC	JNK pathway	[13] Rosa et al., 2016
FRA	GenBank [CK187762]	CTCATCTGTGG AGTTCATGG	GTCAATCACG ACACTAGGAG	JNK pathway	[13] Rosa et al., 2016
Puckered	GenBank [KF828770]	GCGCTTTCATC TGGTGGATC	GCGAAGATCC ATTCCACGAC	JNK pathway	[13] Rosa et al., 2016
JAK	GenBank [KF828771]	CAGAGGTGAT GCTAGTCATG	ATGAGCTGTG GATGAGTGAC	JAK/STAT pathway	[13] Rosa et al., 2016
SOCS	GenBank [KF828775]	CAATCTGGAC CTGAGGGACG	GGAAAGAGGG AACACCAGGG	JAK/STAT pathway	present study
STAT	GenBank [KF828772]	TAGTGTTCCA GGTTTGGACG	CCGTCGCAGA CTTAAACTTC	JAK/STAT pathway	[13] Rosa et al., 2016
PIAS	GenBank [KF828774]	GCATCTACCTC CAGAAGCTC	CATCCTCATCA CTGCTGCAC	JAK/STAT pathway	[13] Rosa et al., 2016
STAM	GenBank [KF828776]	GCATGACGCG AATCCTCGAA	CAGCTCCTGCT CTATCAAGG	JAK/STAT pathway	[13] Rosa et al., 2016
Microplusin	GenBank [AY233212]	CAGTGAAGCC TTCGCATCAG	CCGAAGTCGA AGCCACAAG	Antimicrobial peptide	[14] Capelli-Peixoto et al., 2017
Ixodidin	GenBank [P83516]	CAAAATGCAG TCCCGTTACGT	CCACGACGGC AGAAGCATCC	Antimicrobial peptide	[14] Capelli-Peixoto et al., 2017
Defensin	GenBank [AY233213]	GATGCCCGTTT AACCAAGGA	TTGATTAGGC CAGCGCAGTA	Antimicrobial peptide	[14] Capelli-Peixoto et al., 2017

Caption: MyD88: Mieloid differentiation factor 88; TOLLIP: Toll-interacting protein; SARM: sterile-alpha protein and armadillo motive; Ecsit: evolutionarily conserved signaling intermediate protein in the Toll pathways; TRAF: Factor associated with TNF receptor; NFKIRAS: RAS protein that interacts with IκB; PGRP: peptideoglycan recognition protein; Bendless: protein liga ubiquitin; UEV1a: ubiquitin-conjugating E2 enzyme; EFFETE: ubiquitin-protein ligase; IAP2: apoptosis inhibitor 2; TAK1: transformative growth factor β-1 kinase; TAB2: associated protein 2 to TAK1; Posh: E3 ligase Plenty of SH3; IKK-α: Iκb kinase α; IKK-γ: Iκb kinase γ; Caspar: Factor associated with Fas 1; Caudal: Homeobox CDX-4 protein (caudal-like); RELISH: Transcript factor Rel/NF-κB; .JRA: Jun-related antigen; KAY: Kayak; JAK: Janus kinase; SOCS: suppressor of cytokine signaling; STAT: the transcription factor of the JAK/STAT pathway; PIAS: STAT inhibitory protein; STAM: STAT adapter.

Table S2: Gene expression levels of signaling pathway components in *Rhipicephalus microplus* gut in response to *Theileria equi* infection.

Genes	Experiment 1 (High parasite load)			Experiment 2 (Low parasite load)		
	Fold change mean	*p*-value	IC 95%	Fold change mean	*p*-value	IC 95%
Toll 18Wheeler	4.75	0.31	-6.70 — 3.08	-0.04	0.22	-1.10 — 3.52
Toll	5.07	0.004	-3.09 — -1.50	0.02	0.94	-2.25 —2.37
MyD88	2.69	0.19	-3.83 — 1.11	-0.52	0.28	-3.36 —1.28
TOLLIP	134.73	0.002	-9.20 — -4.86	1.19	0.76	-2.59 — 2.03
Pelle	-0.27	0.84	-4.17 — 4.76	1.76	0.4	-3.11 — 1.53
Pellino	-1.1	0.44	-1.69 — 2.91	2.5	0.34	-3.21 —1.42
Tube	5.41	0.39	-9.23 — 4.50	-0.43	0.79	-2.55 —2.07
SARM	-0.04	0.98	-3.16 —3.21	1.78	0.92	-2.40 —2.22
ECSIT	3.61	0.03	-3.13 — -0.26	-0.62	0.78	-2.55 —2.07
TRAF	11.7	0.09	-7.79 — 0.90	0.31	0.94	-2.39 — 2.25
NFKIRAS	-0.93	0.82	-5.08 — 5.74	-0.54	0.66	-1.92 — 2.72
Cactus (IκB)	-3.42	0.29	-1.61 — 3.87	-0.69	0.45	-1.61 — 3.01
Cactin	7.4	0.15	-6.65 — 2.08	-1.09	0.87	-2.18 — 2.46
Dorsal	1.13	0.47	-3.55 — 2.01	-1.01	0.99	-6.43 — 6.37
PGRP	-1.15	0.77	-5.08 — 6.21	-2.29	0.8	-2.08 — 2.54
Bendless	-0.93	0.72	-3.70 — 4.75	-0.9	0.23	-1.15 — 3.47
UEV1a	-2.21	0.61	-4.34 — 6.39	0.32	0.6	-2.79 — 1.85
EFFETE	2	0.4	-3.79 — 2.00	-0.34	0.7	-2.04 —2.58
IAP2	-0.22	0.96	-5.94 — 6.10	0.83	0.27	-3.38 — 1.24
TAK1	7.46	0.03	-5.01 — -0.59	-1.27	0.88	-2.18 — 2.44
TAB2	-10.5	0.31	-2.22 — 5.24	1	0.14	-0.79 — 3.83
POSH	46.46	0.03	-8.76 — -1.64	-0.54	0.38	-3.12 — 1.50
IKK-α	-2.74	0.53	-2.54 — 4.17	0.75	0.46	-2.99 — 1.65
IKK-γ	-1.72	0.7	-4.88 — 6.21	-0.58	0.22	-1.12 — 3.50
Caspar	7.94	0.41	-5.39 — 2.82	-1.17	0.7	-1.97 — 2.65
Caudal	11.71	0.03	-5.09 — -0.53	0.35	0.44	-0.71 — 0.83
Relish	-3.49	0.48	-3.55 — 5.52	-0.21	0.88	-2.18 — 2.44
Microplusin	0.65	0.5	-8.21 — 4.87	2.50	0.42	-5.01 — 2.70
Hemipterous	-2.36	0.38	-2.11 — 4.26	-0.31	0.98	-2.29 — 2.33
Basket (JNK)	4.65	0.08	-4.78 — 0.37	-0.47	0.73	-2.01 —2.61
JRA	-0.21	0.94	-3.37 — 3.19	-0.55	0.54	-1.76 — 2.88
FRA	2.74	0.22	-4.97 — 2.10	-2.09	0.04	0.19 — 4.83
Puckered	0.84	0.57	-2.82 — 2.01	0.74	0.21	-3.55 — 1.07
JAK	11.98	0.02	-5.95 — -1.19	0.42	0.66	-2.70 — 1.92
STAT	-4.14	0.4	3.63 — 7.00	0.71	0.36	-1.45 — 3.17

PIAS	-0.51	0.54	-1.88 — 2.97	-0.47	0.48	-1.67 — 2.95
SOCS	2.21	0.33	-8.17 — 3.55	-2.7	0.27	-1.28 — 3.45
STAM	-1.04	0.44	-1.99 — 3.23	0.31	0.9	-2.21 — 2.41
Ixodidin	-4.25	0.82	-5.96 — 5.16	1.57	0.52	-3.86 — 2.29
Defensin	10.22	0.08	-8.46 — 0.72	-2.69	0.2	-1.29 — 3.92

Caption: MyD88: Mieloid differentiation factor 88; TOLLIP: Toll-interacting protein; SARM: sterile-alpha protein and armadillo motive; Ecsit: evolutionarily conserved signaling intermediate protein in the Toll pathways; TRAF: Factor associated with TNF receptor; NFKIRAS: RAS protein that interacts with IκB; PGRP: peptideoglycan recognition protein; Bendless: protein liga ubiquitin; UEV1a: ubiquitin-conjugating E2 enzyme; EFFETE: ubiquitin-protein ligase; IAP2: apoptosis inhibitor 2; TAK1: transformative growth factor β-1 kinase; TAB2: associated protein 2 to TAK1; Posh: E3 ligase Plenty of SH3; IKK-α: Iκb kinase α; IKK-γ: Iκb kinase γ; Caspar: Factor associated with Fas 1; Caudal: Homeobox CDX-4 protein (caudal-like); RELISH: Transcript factor Rel/NF-κB; .JRA: Jun-related antigen; KAY: Kayak; JAK: Janus kinase; SOCS: suppressor of cytokine signaling; STAT: the transcription factor of the JAK/STAT pathway; PIAS: STAT inhibitory protein; STAM: STAT adapter.
